# Supplementary material for: ADTnorm: Robust Integration of Single-cell Protein Measurement across CITE-seq Datasets
Source: Res Sq. 2024 Jul 8:rs.3.rs-4572811. Preprint. [Version 1] doi: 10.21203/rs.3.rs-4572811/v1 (PMC11261982; doi:10.21203/rs.3.rs-4572811/v1)
Supplement: Supplement 1 [file NIHPPrs4572811v1-supplement-1.pdf]

Supplementary Table 1: Public CITE-seq data summary

Dataset	Tissue	URL
10X_pbmc_10k	PBMC	https://support.10xgenomics.com/single-cell-gene-expression /datasets/3.0.0/pbmc_10k_protein_v3
10X_pbmc_1k	PBMC	https://www.10xgenomics.com/resources/datasets/1-k-pbm-cs -from-a-healthy-donor-gene-expression-and-cell-surface-protein -3-standard-3-0-0
10X_pbmc_5k_v3	PBMC	https://www.10xgenomics.com/datasets/5k-human-pbmcs-3-v3-1 -chromium-controller-3-1-standard
10X_pbmc_5k_nextgem	PBMC	https://www.10xgenomics.com/resources/datasets/5-k-peripheral -blood-mononuclear-cells-pbm-cs-from-a-healthy-donor-with-cell -surface-proteins-next-gem-3-1-standard-3-1-0
10X_malt_10k	MALT	https://www.10xgenomics.com/resources/datasets/10-k-cells -from-a-malt-tumor-gene-expression-and-cell-surface-protein -3-standard-3-0-0
stuart_2019	Bone Marrow	https://www.ncbi.nlm.nih.gov/geo/query/acc.cgi?acc=GSE128639
granja_2019_bmmc	Bone Marrow	https://www.ncbi.nlm.nih.gov/geo/query/acc.cgi?acc=GSE139369
granja_2019_pbmc	PBMC	https://www.ncbi.nlm.nih.gov/geo/query/acc.cgi?acc=GSE139369
hao_2020	PBMC	https://atlas.fredhutch.org/nygc/multimodal-pbmc/
kotliarov_2020	PBMC	https://nih.figshare.com/articles/dataset/CITE-seq_protein-mRNA _single_cell_data_from_high_and_low_vaccine_responders_to _reproduce_Figs_4-6_and_associated_Extended_Data_Figs_ /11349761
witkowski_2020	Bone Marrow	https://www.ncbi.nlm.nih.gov/geo/query/acc.cgi?acc=GSE153358
triana_2021	Bone Marrow	https://figshare.com/projects/Single-cell_proteo-genomic_reference _maps_of_the_human_hematopoietic_system/94469
buus_2021_T	Only keep T cells	https://figshare.com/collections/Improving_oligo-conjugated _antibody_signal_in_multimodal_single-cell_analysis/5018987
Nettersheim_2022	PBMC (Titration)	https://www.ncbi.nlm.nih.gov/geo/query/acc.cgi?acc=GSE213282
Stephenson_2021	PBMC (COVID-19)	https://www.ebi.ac.uk/biostudies/arrayexpress/studies/E-MTAB-10026

*PBMC: Peripheral Blood Mononuclear Cells; MALT: Mucosa-Associated Lymphoid Tissue.

Supplementary Table 2: Manual gating strategy.

Cell Type	Gating Strategy
B	$CD3^{-}CD19^{+}$
CD4 T	$CD3^{+}CD19^{-}CD4^{+}CD8^{-}$
CD8 T	$CD3^{+}CD19^{-}CD4^{-}CD8^{+}$
DC	$CD3^{-}CD19^{-}CD20^{-}CD14^{-}HLA\text{-}DR^{+}CD56^{-}CD16^{-}$
NK	$CD3^{-}CD19^{-}CD20^{-}CD14^{-}HLA\text{-}DR^{-}CD56^{+}$
Monocytes	$CD3^{-}CD19^{-}CD20^{-}CD14^{+}$
naïve B	$CD3^{-}CD19^{+}CD27^{-}$
memory B	$CD3^{-}CD19^{+}CD27^{+}$
naive CD4	$CD3^{+}CD19^{-}CD4^{+}CD8^{-}CD25^{-}CD45RA^{+}CD45RO^{-}$
memory CD4	$CD3^{+}CD19^{-}CD4^{+}CD8^{-}CD25^{-}CD45RA^{-}CD45RO^{+}$
Treg	$CD3^{+}CD19^{-}CD4^{+}CD8^{-}CD25^{+}CD127^{-}$
naive CD8	$CD3^{+}CD19^{-}CD4^{-}CD8^{+}CD45RA^{+}CD45RO^{-}$
memory CD8	$CD3^{+}CD19^{-}CD4^{-}CD8^{+}CD45RA^{-}CD45RO^{+}$
plasmacytoid DC	$CD3^{-}CD19^{-}CD20^{-}CD14^{-}HLA\text{-}DR^{+}CD56^{-}CD16^{-}CD123^{+}CD11c^{-}$
myeloid DC	$CD3^{-}CD19^{-}CD20^{-}CD14^{-}HLA\text{-}DR^{+}CD56^{-}CD16^{-}CD123^{-}CD11c^{+}$
$CD16^{+}$ NK	$CD3^{-}CD19^{-}CD20^{-}CD14^{-}HLA\text{-}DR^{-}CD56^{+}CD16^{+}$
$CD16^{-}$ NK	$CD3^{-}CD19^{-}CD20^{-}CD14^{-}HLA\text{-}DR^{-}CD56^{+}CD16^{-}$
classical monocyte	$CD3^{-}CD19^{-}CD20^{-}CD14^{+}CD16^{-}$
intermediate monocyte	$CD3^{-}CD19^{-}CD20^{-}CD14^{+}CD16^{+}$
non-classical $CD16^{+}$ monocyte	$CD3^{-}CD19^{-}CD20^{-}CD14^{-}HLA\text{-}DR^{+}CD56^{-}CD16^{+}$
